# Supplementary material for: Introduction of Nonacidic Side Chains on 6-Ethylcholane Scaffolds in the Identification of Potent Bile Acid Receptor Agonists with Improved Pharmacokinetic Properties
Source: Molecules. 2019 Mar 16;24(6):1043. doi: 10.3390/molecules24061043 (PMC6470523; doi:10.3390/molecules24061043)

Supplementary material

Introduction of nonacidic side chains on 6-ethylcholane scaffolds in the identification of potent bile acid receptor agonists with improved pharmacokinetic properties

Claudia Finamore [1], Giuliana Baronissi [1], Silvia Marchianò [2], Francesco Saverio Di Leva [1], Adriana Carino [2], Maria Chiara Monti [4], Vittorio Limongelli [1,3], Angela Zampella [1], Stefano Fiorucci [2] and Valentina Sepe [1*]

[1]Department of Pharmacy, University of Naples "Federico II", via D. Montesano 49, 80131 Naples, Italy.
[2]Department of Surgery and Biomedical Sciences, Nuova Facoltà di Medicina, Perugia, Italy
[3]Università della Svizzera Italiana (USI), Faculty of Biomedical Sciences, Institute of Computational Science - Center for Computational Medicine in Cardiology, Via G. Buffi 13, CH-6900 Lugano, Switzerland.
[4]Department of Pharmacy, University of Salerno, Via Giovanni Paolo II, 132, 84084, Fisciano, Salerno, Italy.

Table of contents:

Figure S1. Docking pose of **1** (A) and **6** (B) at the FXR ligand binding domain S2
Figure S2. Docking pose of **1** in the GPBAR1 homology model S2
Figure S3 and S4. ^{1}H-NMR and ^{13}C-NMR spectra of compound **1** S3
Figure S5 and S6. ^{1}H-NMR and ^{13}C-NMR spectra of compound **2** S4
Figure S7 and S8. ^{1}H-NMR and ^{13}C-NMR spectra of compound **3** S5
Figure S9 and S10. ^{1}H-NMR and ^{13}C-NMR spectra of compound **4** S6
Figure S11 and S12. ^{1}H-NMR and ^{13}C-NMR spectra of compound **5** S7
Figure S13 and S14. ^{1}H-NMR and ^{13}C-NMR spectra of compound **6** S8
Figure S15 and S16. ^{1}H-NMR and ^{13}C-NMR spectra of compound **8** S9
Figure S17 and S18. ^{1}H-NMR and ^{13}C-NMR spectra of compound **10** S10

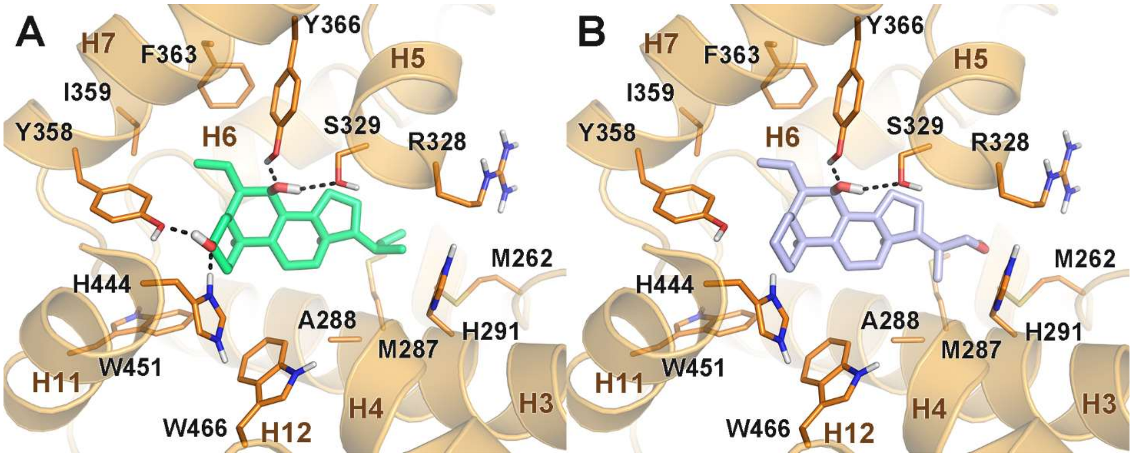

Figure S1. Docking pose of **1** (A) and **6** (B) at the FXR ligand binding domain. Compounds **1** and **6** are depicted as green and light blue sticks, respectively. FXR is shown as orange cartoons. Amino acids important for ligand binding are shown as sticks. Non-polar hydrogens are omitted for clarity. Hydrogen bonds are shown as dashed black lines.

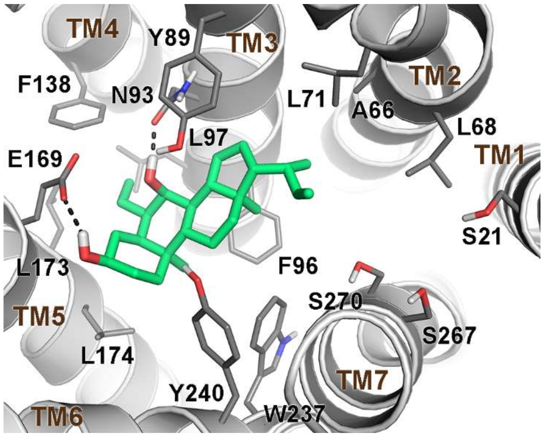

Figure S2. Docking pose of **1** in the GPBAR1 homology model.[3] Compound **1** is depicted as green sticks, while GPBAR1 is shown as grey cartoons. Amino acids important for ligand binding are shown as sticks. Non-polar hydrogens are omitted for clarity. Hydrogen bonds are shown as dashed black lines.

Figure S3. ^{1}H NMR (400 MHz, $CDCl_3$) of compound **1**

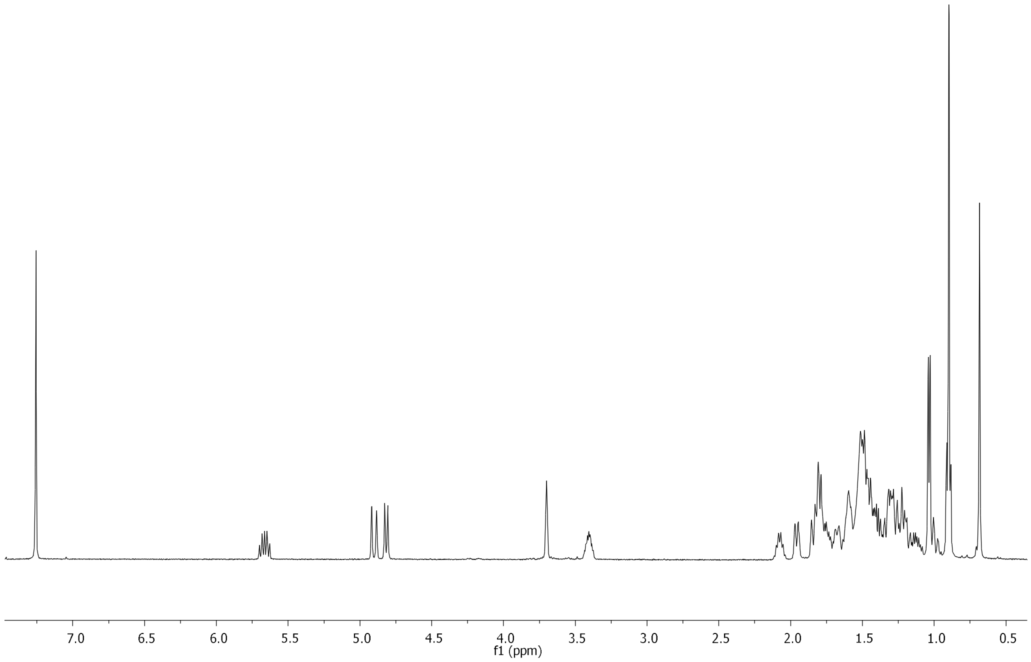

Figure S4. ^{13}C NMR (100 MHz, $CDCl_3$) of compound **1**

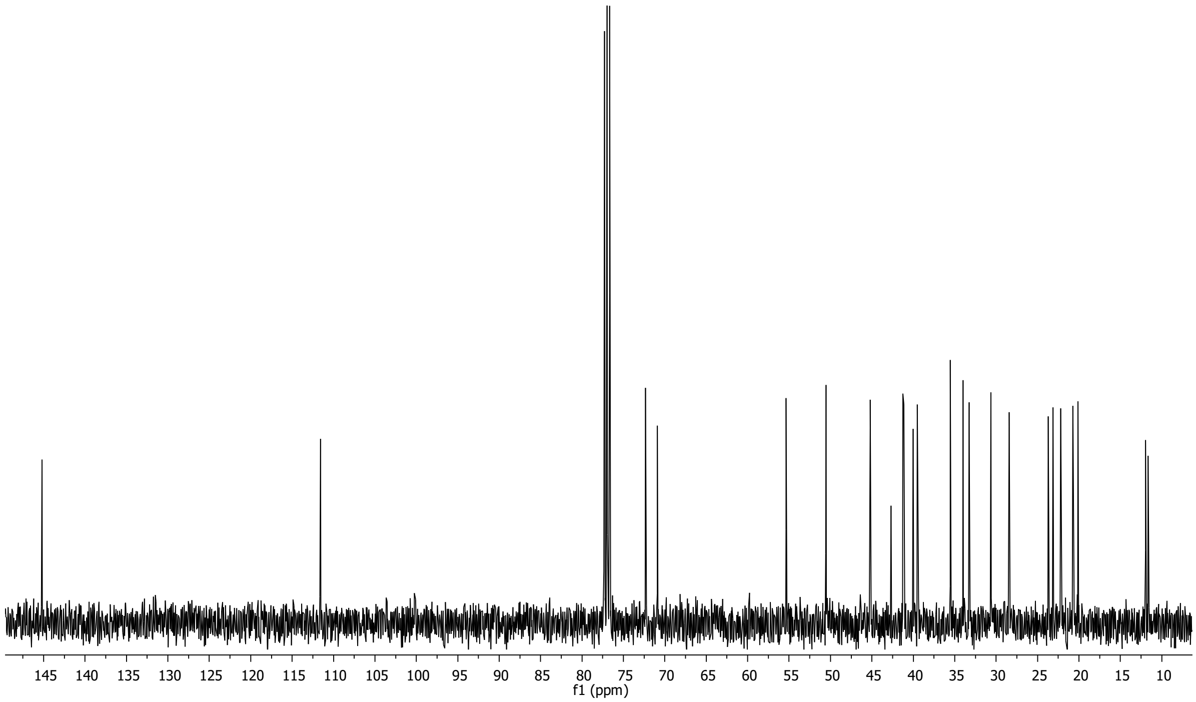

Figure S5. ^{1}H NMR (400 MHz, CD_3OD) of compound **2**

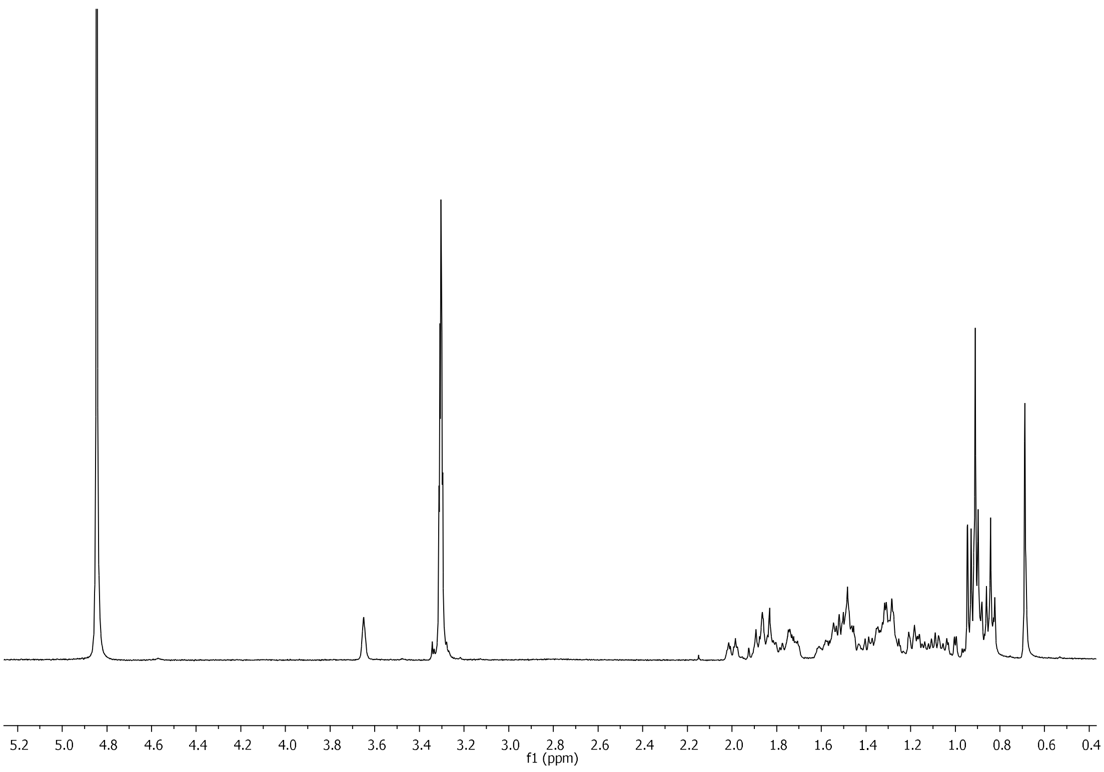

Figure S6. ^{13}C NMR (100 MHz, CD_3OD) of compound **2**

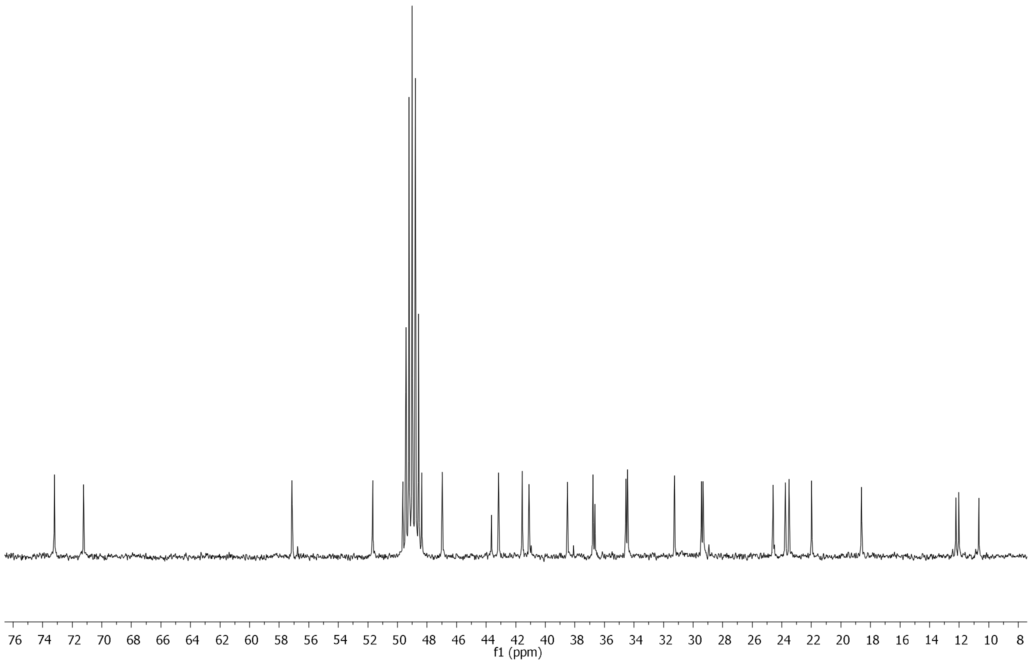

Figure S7. ^{1}H NMR (400 MHz, $CDCl_3$) of compound **3**

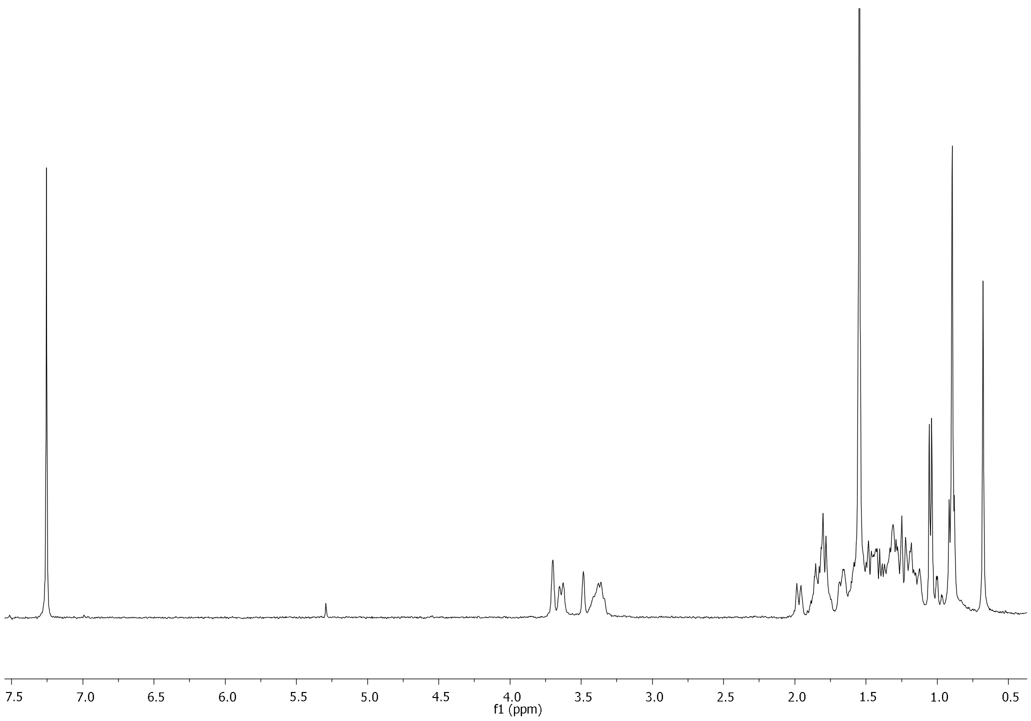

Figure S8. ^{13}C NMR (100 MHz, $CDCl_3$) of compound **3**

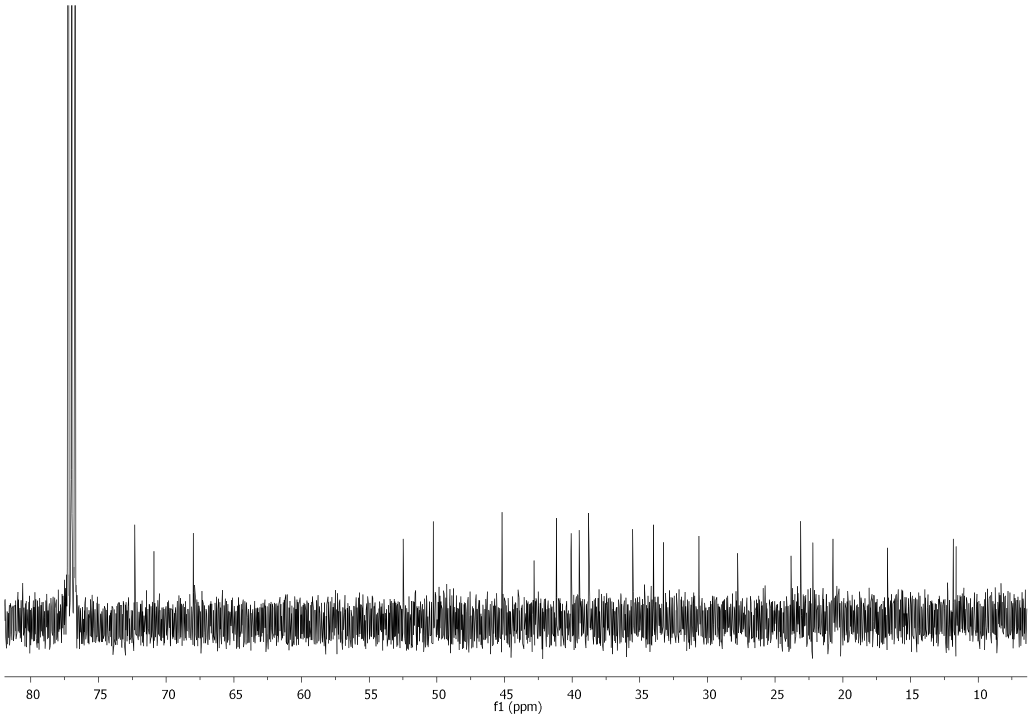

Figure S9. ^{1}H NMR (400 MHz, $CDCl_3$) of compound **4**

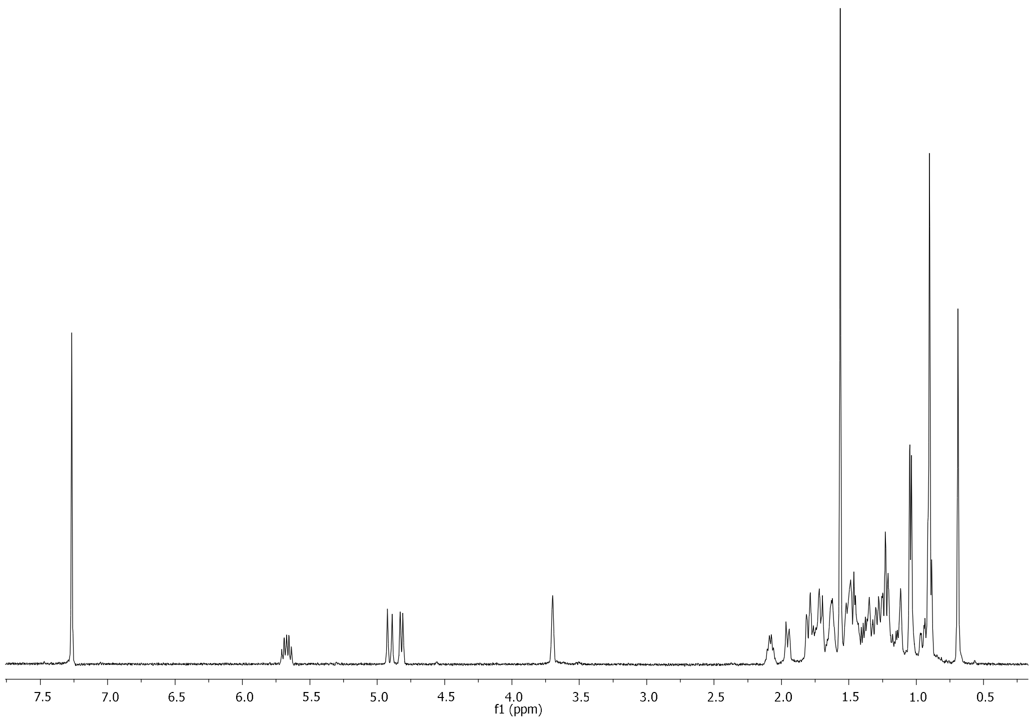

Figure S10. ^{13}C NMR (100 MHz, $CDCl_3$) of compound **4**

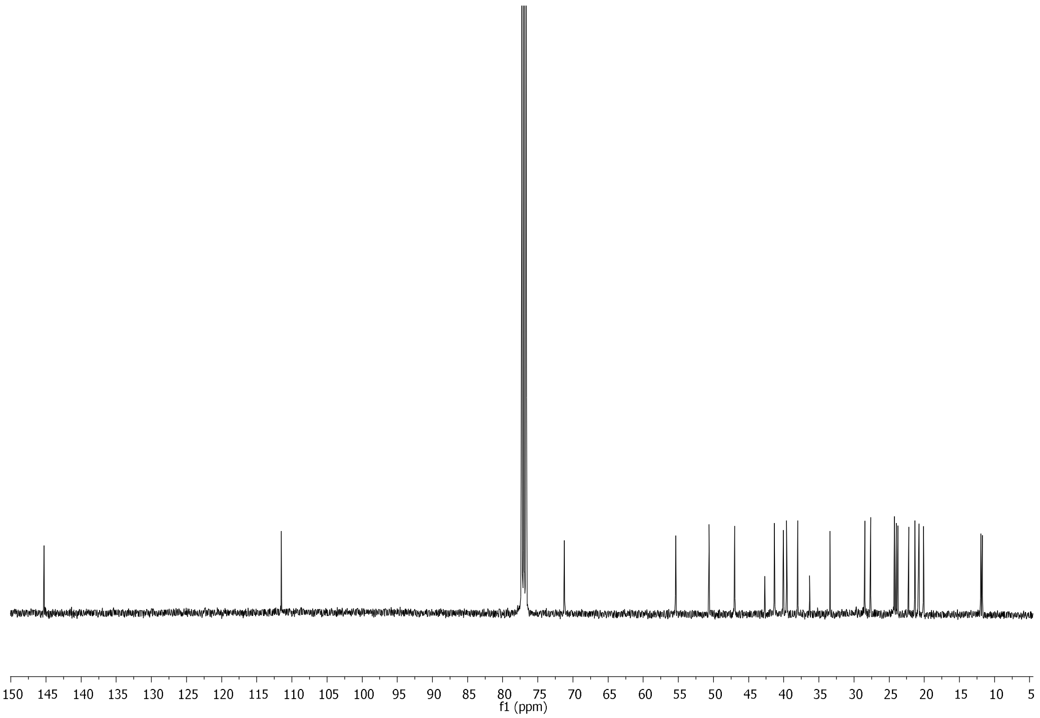

Figure S11. ^{1}H NMR (400 MHz, $CDCl_3$) of compound **5**

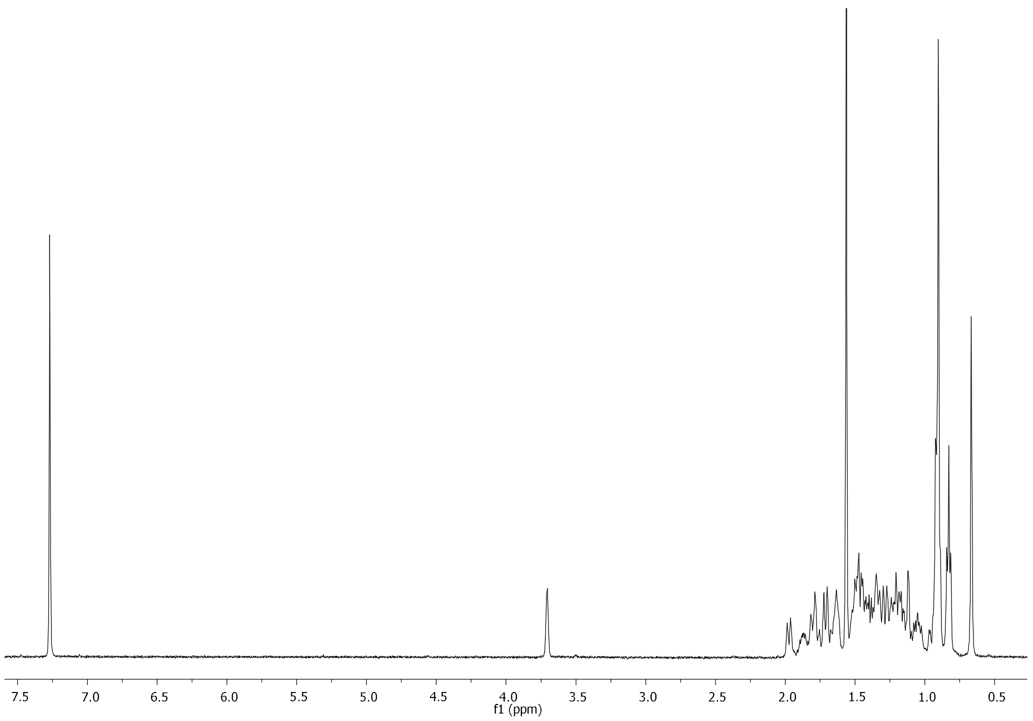

Figure S12. ^{13}C NMR (100 MHz, $CDCl_3$) of compound **5**

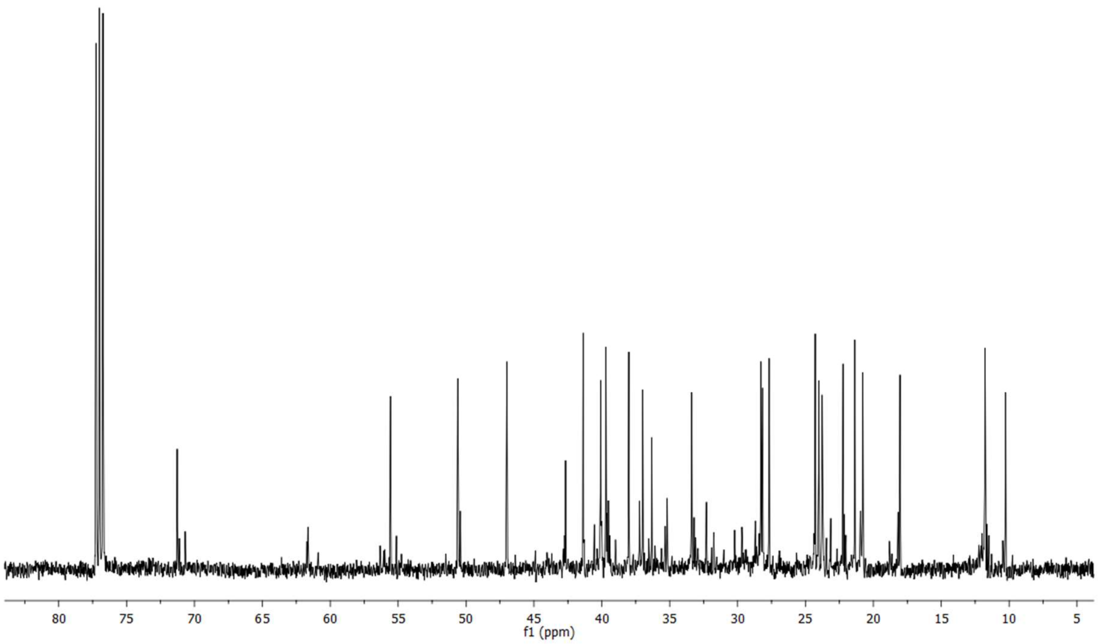

Figure S13. ^{1}H NMR (400 MHz, $CDCl_3$) of compound **6**

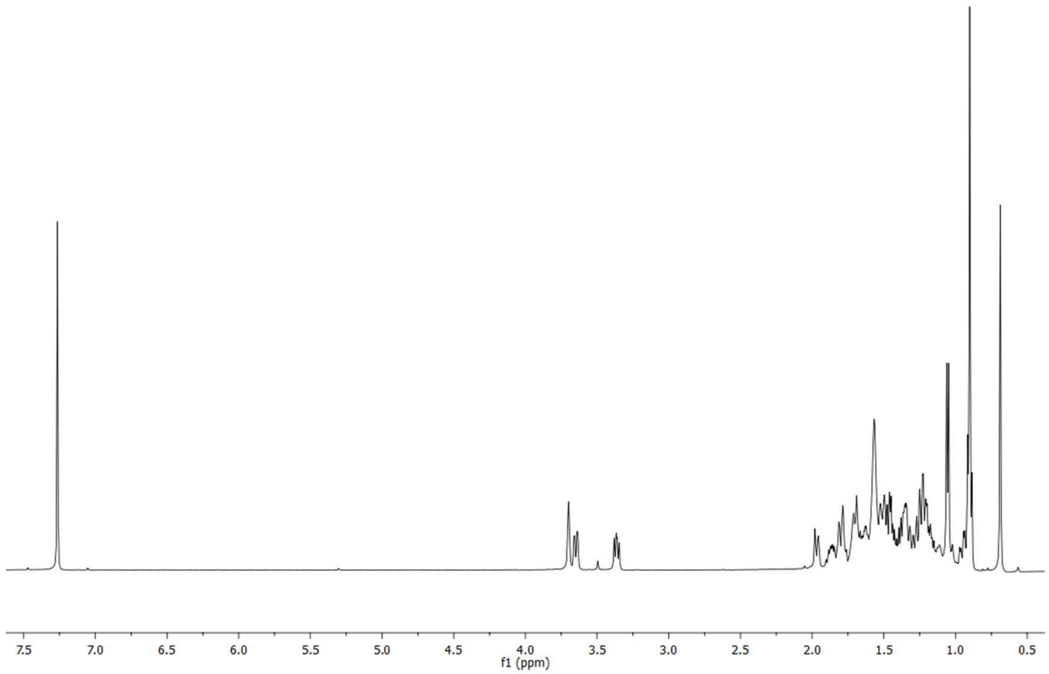

Figure S14. ^{13}C NMR (100 MHz, $CDCl_3$) of compound **6**

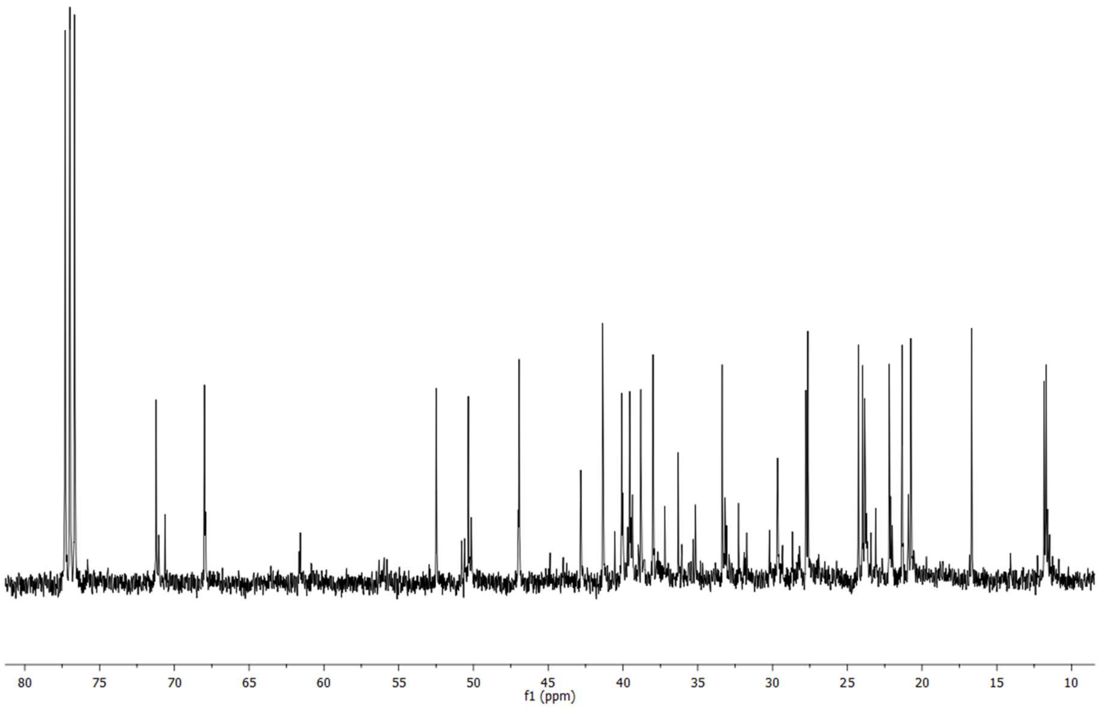

Figure S15. ^{1}H NMR (400 MHz, $CDCl_3$) of compound **8**

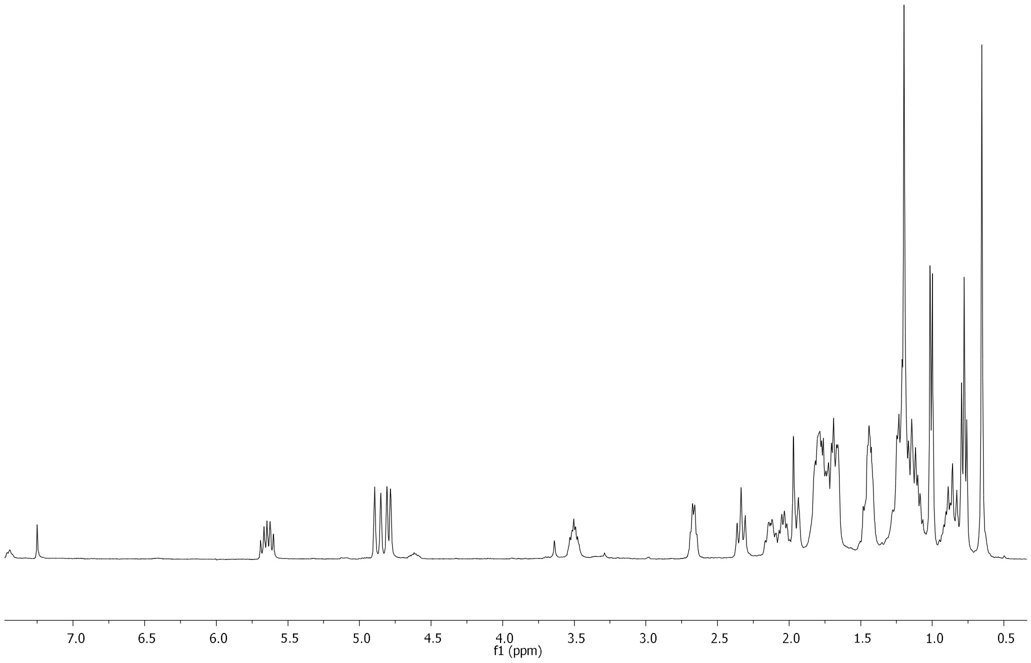

Figure S16. ^{13}C NMR (100 MHz, $CDCl_3$) of compound **8**

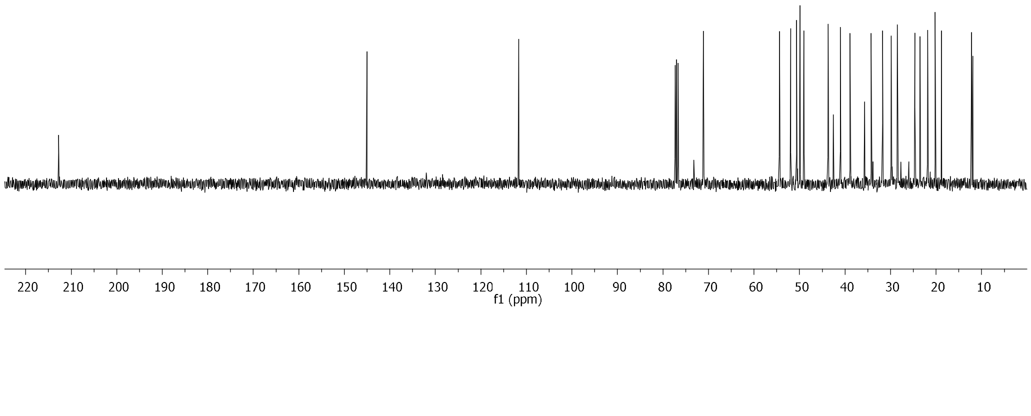

Figure S17. ^{1}H NMR (400 MHz, CD_3OD) of compound **10**

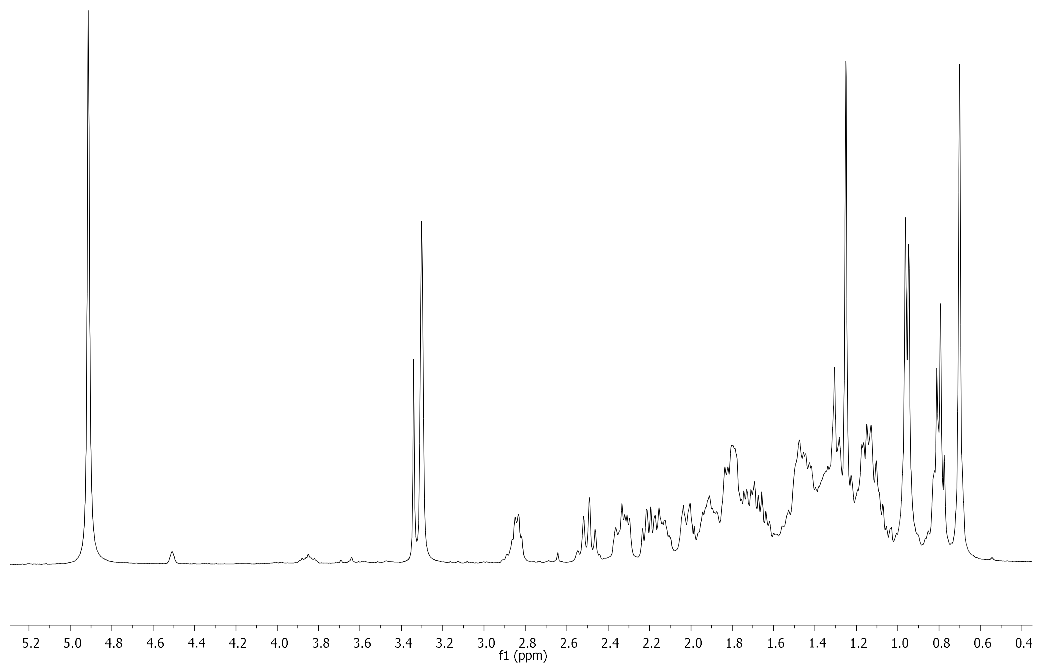

Figure S18. ^{13}C NMR (100 MHz, $CDCl_3$) of compound **10**

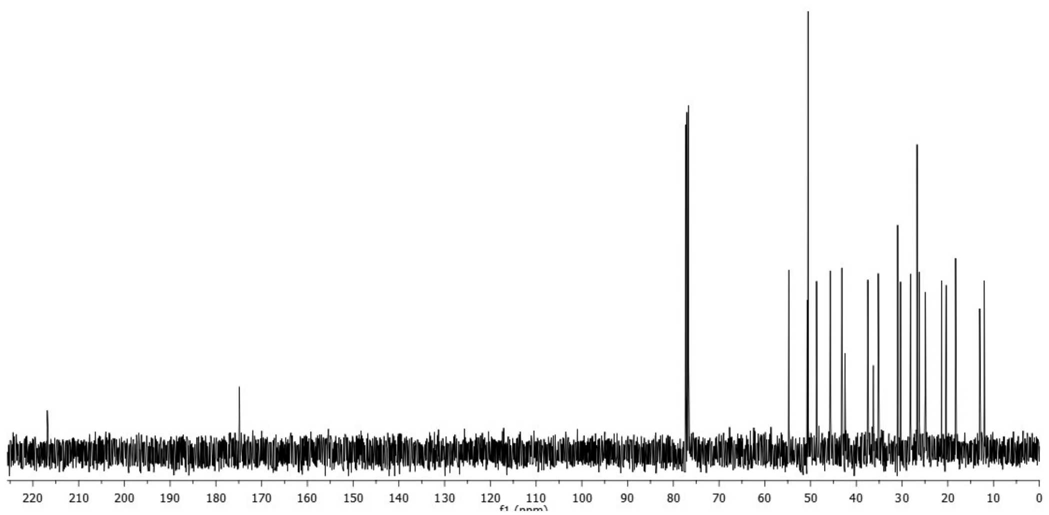

Supplement: Supplementary file 1 [file molecules-24-01043-s001.pdf]
